# Pharmacokinetics of Ziyuglycoside I and Ziyuglycoside II in Rat Plasma by UPLC-MS/MS

**DOI:** 10.1155/2024/7971021

**Published:** 2024-03-01

**Authors:** Xiuwei Shen, Ziyue Wang, Wenting Li, Shenshen Mei, Shunjun Ma, Xianqin Wang, Congcong Wen, Fan Chen, Guojun Zheng

**Affiliations:** ^1^Ruian People's Hospital, The Third Affiliated Hospital of Wenzhou Medical University, Wenzhou, China; ^2^Laboratory Animal Centre, Wenzhou Medical University, Wenzhou, China; ^3^School of Pharmaceutical Sciences, Wenzhou Medical University, Wenzhou, China

## Abstract

Ziyuglycoside I and ziyuglycoside II are important active components of *Sanguisorba officinalis* L., which have excellent pharmacological effects, such as antioxidant and anticancer effects. However, the bioavailability of ziyuglycoside I and ziyuglycoside II has not been reported. This work aims to establish a UPLC-MS/MS method to study the pharmacokinetics of ziyuglycoside I and ziyuglycoside II in rats under different administration routes (intragastric and intravenous administration) and to calculate the bioavailability. The concentration of ziyuglycoside I and ziyuglycoside II in rat plasma in the range of 2–2000 ng/mL showed a good linear relationship (*r* > 0.99). The intra-day accuracies of ziyuglycoside I and ziyuglycoside II ranged from 87% to 110%, and the inter-day accuracies ranged from 97% to 109%. The intra-day precision was less than 15% and the inter-day precision was less than 14%. The matrix effects ranged from 88% to 113%. The recoveries were all above 84%. The developed UPLC-MS/MS method for the determination of ziyuglycoside I and ziyuglycoside II in rat plasma was applied to pharmacokinetics. The bioavailability of ziyuglycoside I and ziyuglycoside II was measured at 2.6% and 4.6%, respectively.

## 1. Introduction

*Sanguisorba officinalis* L. belongs to the genus *Sanguisorba* in the family Rosaceae [1–3]. It has long been used as a natural remedy for inflammatory and metabolic diseases, such as chronic intestinal infections, bleeding, diarrhea, and duodenal ulcer [4–6]. Ziyuglycoside I and ziyuglycoside II, isolated from *Sanguisorba officinalis* L., are the important active components of *Sanguisorba officinalis* L., which have excellent pharmacological effects. Many studies have shown that saponins extracted from *Sanguisorba officinalis* L. have antioxidant and anticancer effects [7–9]. Among other things, ziyuglycoside I could prevent the formation of collagen fibers, effectively promote the production of type I collagen, and reduce the generation of skin wrinkles, which could be used in the synthesis of beauty and skin care products [7, 10]. Ziyuglycoside II could improve diabetes performance and protect liver and kidney [11].

To date, some studies on the pharmacokinetics of ziyuglycoside I and ziyuglycoside II using LC-MS/MS have been published [12, 13]. Li et al. developed an HPLC-MS/MS method for the quantification of ziyuglycoside I and ziyuglycoside II in rat biological matrices, with a total run time of 6 min for a sample and a lower limit of quantification (LLOQ) of 2 ng/mL [12], and the pharmacokinetics, tissue distribution, and excretion of ziyuglycoside I and ziyuglycoside II were evaluated. Ye et al. developed an LC-MS/MS method for the determination of ziyuglycoside I and ziyuglycoside II in rat plasma using liquid-liquid extraction with n-butanol. Chromatographic separation was achieved using a Thermo Golden C 18 column with a total run time of 10 min for a sample and LLOQ of 2 ng/mL [13]. Wu et al. developed a UHPLC-MS/MS method for determination and pharmacokinetic study of six triterpenes in rat plasma after oral administration of *Sanguisorba officinalis* L. extract [14]. However, only ziyuglycoside I was determined with an LLOQ of 6.05 ng/mL, and ziyuglycoside II was not determined. In addition, these studies on the bioavailability of ziyuglycoside I and ziyuglycoside II have not been published. Therefore, it is necessary to establish a simple and sensitive UPLC-MS/MS method for determining the bioavailability of ziyuglycoside I and ziyuglycoside II.

The aim of this work is to establish a UPLC-MS/MS method to investigate the pharmacokinetics of ziyuglycoside I and ziyuglycoside II in rats under different routes of administration (intragastric and intravenous administration) and calculate the bioavailability. It is expected to help in the determination of plasma concentration of ziyuglycoside I and ziyuglycoside II, development and research of new ziyuglycoside drugs, and toxicology research.

## 2. Experimental

### 2.1. Chemicals

**Figure 1:**
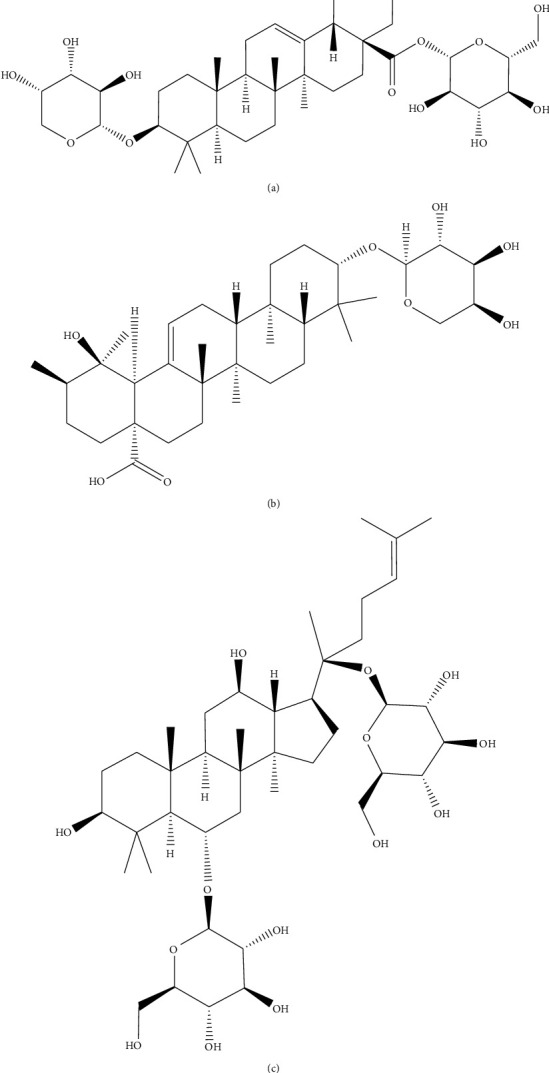
Chemical structures of ziyuglycoside I (a), ziyuglycoside II (b), and ginsenoside Rg1 (c).

Ziyuglycoside I, ziyuglycoside II, and ginsenoside Rg1 (purity >98%, Figure 1) were purchased from Chengdu Must Biotechnology Co., LTD., China. HPLC-grade acetonitrile, methanol, and formic acid were purchased from Merck, Germany. Ultrapure water was manufactured by Milli-Q Water Systems, USA.

### 2.2. Apparatus and Conditions

ACQUITY H-Class UPLC system and Xevo TQS-Micro triple quadrupole mass spectrometer (Waters Corp, Milford, MA, USA) were used in this work.

A UPLC HSS T3 column (2.1 mm × 50 mm, 1.8 *μ*m) was used with gradient elution at a flow rate of 0.4 mL/min with a mobile phase of acetonitrile-water (containing 0.1% formic acid). The gradient elution conditions were as follows: 0–0.2 min, acetonitrile 10%; 0.2–1.2 min, acetonitrile 10%–90%; 1.2–2.0 min, acetonitrile 90%–90%; 2.0–2.2 min, acetonitrile 90%–10%; and 2.2–3.5 min, acetonitrile 10%.

Nitrogen was used as the desolvation gas (926 L/h) and atomization gas with a capillary voltage of 2.06 kV and an ion source temperature of 147°C, and the desolvation temperature was 499°C. The positive and negative ion mode of ESI was simultaneous monitoring. The positive ion mode of ESI was used for ziyuglycoside II, and the negative ion mode of ESI used for ziyuglycoside I and ginsenoside Rg1. The MRM mode was used for quantitative analysis, m/z 811.291→603.379 was for ziyuglycoside I (cone voltage 34 v and collision voltage 28 v), m/z 605.37→455.36 was for ziyuglycoside II (cone voltage 14 v and collision voltage 28 V), and m/z 845.426→637.529 was for internal standard ginsenoside Rg1 (cone voltage 4 v and collision voltage 28 v).

### 2.3. Preparation of Reference Solution

5.0 mg of ziyuglycoside I, ziyuglycoside II, and ginsenoside Rg1 was accurately weighed and transferred to a 10 mL volumetric flask and diluted with methanol to the scale line to make a concentration of 0.50 mg/mL, respectively. The working solution of ginsenoside Rg1 was then transferred to a reagent bottle and diluted to 100 mL with methanol at a concentration of 1.0 *μ*g/mL. A series of working solutions were prepared by diluting the stock solution of ziyuglycoside II (0.50 mg/mL) and ziyuglycoside I (0.50 mg/mL) with methanol. All solutions were stored in a refrigerator at −20°C and returned to room temperature before use.

### 2.4. Preparation of Standard Curves

The ziyuglycoside I and ziyuglycoside II series standards were prepared by adding a suitable working solution to the blank plasma. The standard curve was 2000, 1000, 500, 200, 100, 50, 20, 10, 5, and 2 ng/mL, respectively. Quality control (QC) samples with plasma concentrations (2, 8, 150, and 1500 ng/mL) were prepared using the same method.

### 2.5. Plasma Sample Pretreatment

The plasma sample was pretreated by liquid-liquid extraction, that is, 100 *µ*L of plasma sample and 20 *µ*L of internal standard (1.0 *μ*g/mL) were added to a 1.5 mL centrifuge tube, vortexed, and mixed for 30 s, and then 0.9 mL ethyl acetate was added for extraction. The samples were then centrifuged at 12000 rpm for 10 min, and the organic phase was transferred to another centrifuge tube and blow-dried at 55°C under a stream of nitrogen. 100 *µ*L of methanol was added to the blown dried centrifuge tube, vortexed, and mixed again, and a small amount of the mixed solution was placed in a bottle for detection.

### 2.6. Method Validation

The validation method was established in accordance with the US Food and Drug Administration (FDA) Bioanalytical Method Validation Guidelines [15]. Validation criteria included selectivity, linearity, precision, accuracy, matrix effect, recovery, and stability [16, 17].

### 2.7. Pharmacokinetics

Sprague Dawley (SD) rats (male, weighing 220–250 g) were purchased from the Animal Experiment Center of Wenzhou Medical University. Before the experiment, food intake was prohibited for 12 hours, but water was freely available. All experimental procedures were approved by the Animal Care Committee of Wenzhou Medical University (wydw2023-0576). 24 rats were divided into 4 groups with 6 rats in each group. In groups 1 and 2, the rats were administered ziyuglycoside I at a dose of 1 mg/kg intravenously (iv) and 5 mg/kg by gavage (ig), respectively. Intravenous administration is administered via sublingual vein. In groups 3 and 4, the rats were administered ziyuglycoside II at a dose of 1 mg/kg intravenously (iv) and 5 mg/kg by gavage (ig), respectively. At 0.0833, 0.25, 1, 2, 4, 6, 8, 12, and 24 h, 0.3 mL of tail vein blood was collected into heparinized tubes and centrifuged at 13,000 rpm for 10 min. Subsequently, 100 *µ*L of the top layer plasma was transferred to a new 1.5 mL centrifuge tube, the corresponding administration times were marked on the centrifuge tube, and it was stored at −20°C. Pharmacokinetic data were determined using DAS (Drug and Statistics) 2.0 software (Shanghai University of Traditional Chinese Medicine, China).

## 3. Results and Discussion

### 3.1. Methodology Development

To obtain the best conditions for mass spectrometry, positive ion mode and negative ion mode were used for monitoring [16, 18]. The reactivity of ziyuglycoside I in negative ion mode was higher and the reactivity of ziyuglycoside II in positive ion mode was higher. After optimizing various parameters, the capillary tension and collision energy were finally determined.

To select the appropriate plasma processing method, the extraction efficiencies of analytes by liquid-liquid extraction, solid-liquid extraction, and protein precipitation methods were studied and compared [19]. The solid-liquid extraction method offers better results in extraction yield and matrix effect, but at the expense of higher cost and operational complexity. Using the protein precipitation method, plasma samples could be processed easily and quickly [20], but the extraction efficiency was not high. Liquid-liquid extraction exhibits commendable selectivity [21], minimal matrix effect, and satisfactory extraction efficiency. Ethyl acetate has a lower boiling point than n-butanol and was more volatile, making it more suitable for liquid-liquid extraction. Due to the detection sensitivity requirements, the ethyl acetate liquid-liquid extraction method was selected to process the plasma samples in this work.

**Figure 2:**
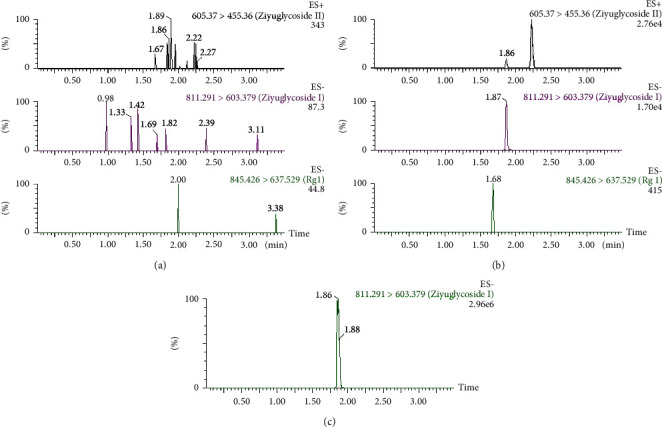
UPLC-MS/MS of ziyuglycoside I, ziyuglycoside II, and ginsenoside Rg1 in rat plasma. (a) Blank rat plasma. (b) Blank rat plasma spiked with ziyuglycoside I, ziyuglycoside II, and ginsenoside Rg1. (c) A sample after intravenous administration of ziyuglycoside I.

The elution systems consisting of methanol, acetonitrile, water, and formate water were compared according to the physicochemical properties and the chromatographic behavior of the analytes [20, 22–24]. The results show that the separation effect of methanol-water or acidic water as system components was not ideal, but the optimized separation effect of acetonitrile-water-formic acid system components was better, and each chromatographic peak separation and the theoretical number of trays met the requirements. In addition, the retention time of the analytes can be changed by selecting the gradient elution conditions, so that the matrix effect can be reduced by separating the analytes from the co-effluent. Only 4 min was needed for a sample, and it was faster than 6 min and 7.5 min as reported in the literature [12, 13]. The retention times of ziyuglycoside I, ziyuglycoside II, and ginsenoside Rg1 were 1.87, 2.24, and 1.68 min using acetonitrile-water-formic acid system as shown in Figure 2, respectively.

In the quantitative analysis of biological samples, the deuterated standard was the best internal standard to correct for loss in the extraction process [25]. However, this internal standard was expensive and not always available for purchase. Therefore, it was preferred to select a compound with similar structure, recovery, and mass spectrometric ion response as the analyte. Ginsenoside Rg1 was chosen as the internal standard in this study because its polarity and ion response corresponded to those of ziyuglycoside I and ziyuglycoside II.

### 3.2. Method Validation

The selectivity of the method was assessed by analyzing blank rat plasma and blank plasma spiked with ziyuglycoside I and II as well as an internal standard. Figure 2 shows the UPLC-MS/MS chromatogram of blank rat plasma and blank rat plasma spiked with ziyuglycoside I and ziyuglycoside II. There was no influence of endogenous substances on the determination of the analytes.

The calibration curves were generated by analyzing spiked calibration samples on three different days. The resulting standard curves were well-fitted to the equations through linear regression, employing a weighting factor of the reciprocal of the concentration (1/*x*). The lower limit of quantification (LLOQ) was defined as the minimum concentration observed on the calibration curves. The concentration of ziyuglycoside I and ziyuglycoside II in rat plasma in the range of 2–2000 ng/mL showed a linear relationship, and the standard curve equations were *y* = 0.3642*x* + 1.0438, *r* = 0.9976; *y* = 0.029*x* + 0.1111, *r* = 0.9981; y represents the peak area of ziyuglycoside I and ziyuglycoside II, and *x* represents the concentration of ziyuglycoside I and ziyuglycoside II in plasma. The LLOQ of ziyuglycoside I and ziyuglycoside II in rat plasma was 2 ng/mL. The limit of detection (LOD), defined as a signal-to-noise ratio of 3, of ziyuglycoside I and ziyuglycoside II in rat plasma was 0.5 ng/mL.

The accuracy and precision of the assay were evaluated by analyzing quality control (QC) samples at three different concentration levels in six replicates over a three-day validation period. Precision was quantified as relative standard deviation (RSD). As presented in Table 1, the intra-day accuracies for ziyuglycoside I and ziyuglycoside II ranged from 87% to 110%, while the inter-day accuracies ranged from 97% to 109%. The intra-day precision was below 15%, and the inter-day precision was below 14%.

To assess the matrix effect, rat blank plasma was extracted and supplemented with ziyuglycoside I and ziyuglycoside II at concentrations of 2, 8, 150, and 1500 ng/ml (*n* = 6). Subsequently, the corresponding peak areas were compared to those obtained from pure standard solutions at equivalent concentrations. The observed matrix effects ranged from 88% to 113% (Table 1).

The recovery of both ziyuglycoside I and ziyuglycoside II was evaluated by comparing the peak area of extracted quality control samples with that of reference quality control solutions reconstituted in blank plasma extracts (*n* = 6). All recoveries exceeded 84% (Table 1).

The stability values of ziyuglycoside I and ziyuglycoside II in rat plasma were assessed by analyzing three replicates of quality control (QC) plasma samples, each exposed to distinct conditions. Specifically, the rat plasma samples underwent pretreatment and were stored at room temperature for 24 h, subjected to three freeze-thaw cycles, and their stability was further examined during long-termstorage at −20°C. The results demonstrated an accuracy range of 92%–108% and an RSD within 13%, indicating acceptable stability of both ziyuglycoside I and ziyuglycoside II.

### 3.3. Pharmacokinetics Study

**Figure 3:**
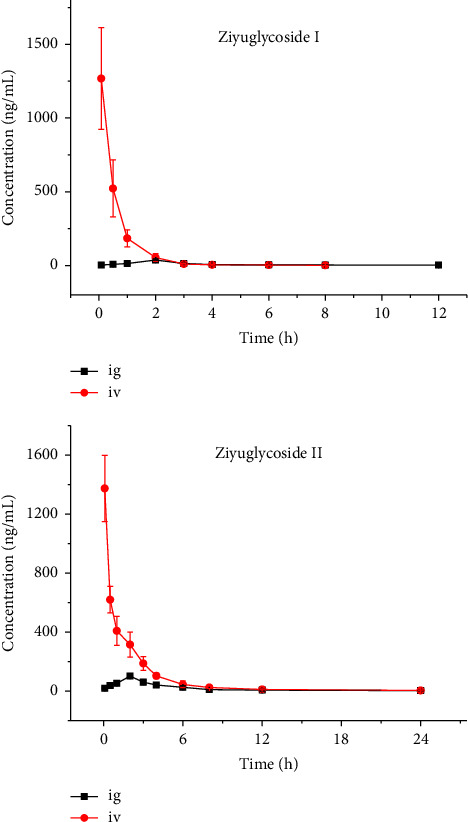
The concentration-time curve of rats after intravenous (iv, 1 mg/kg) and intragastric (ig, 5 mg/kg) administration of ziyuglycoside I and ziyuglycoside II.

The plasma concentration-time curve, with the administration time as the *x*-axis and the plasma concentration as the *y*-axis, is depicted in Figure 3. Pharmacokinetic parameters were determined using a noncompartmental model, and the corresponding data are presented in Table 2. Following intragastric administration of ziyuglycoside I, the half-life (*t*_1/2_), area under the curve from time zero to infinity (AUC_(0-∞)_), and clearance (CL) were calculated to be 5.1 ± 2.5 h, 109.0 ± 11.8 ng/mL *∗* h, and 46.3 ± 5.2 L/h/kg, respectively. For intravenous administration of ziyuglycoside I, *t*_1/2_ was found to be 1.8 ± 0.7 h while AUC_(0-∞)_ and CL were measured as 838.3 ± 250 0.3 ng/mL *∗* h and 1 0.3 ± 0.3 L/h/kg, respectively. The reported values for *t*_1/2_ after tail vein administration (1.338 ± 0.744 h) and subcutaneous injection administration (6.115 ± 1.92 h) [12] were consistent with our findings. The observed *t*_1/2_ value of 19.76 ± 1.59 h for ziyuglycoside I following oral administration of *Sanguisorba officinalis* L. extract at a dose of 2.0 g/kg to rats [13] was not congruent with our results.

*t*_1/2_, AUC_(0-∞)_, and CL of ziyuglycoside II following intragastric administration were determined as 4.9 ± 1.5 h, 458.3 ± 46.3 ng/mL *∗* h, and 11.0 ± 1.0 L/h/kg, respectively. For intravenous administration, *t*_1/2_ of ziyuglycoside II was found to be 6.2 ± 3.1 h, AUC_(0-∞)_ was measured as 1979.2 ± 185.7 ng/mL *∗* h, and CL was calculated as being equal to 0.5 L/h/kg. The literature reports a *t*_1/2_ of ziyuglycoside II after tail vein administration and subcutaneous injection as being equal to approximately 1.027 ± 0.057 h and 7.935 ± 3.264 h [12]. Furthermore, it is reported that *t*_1/2_ of ziyuglycoside II after oral administration of *Sanguisorba officinalis* L. extract is approximately 12.16 ± 4.44 h which differs from our results. However, no information regarding the bioavailability of both ziyuglycoside I and ziyuglycoside II has been provided in these literature sources [12, 13]. In this study, we have determined that the bioavailability values for ziyuglycoside I and ziyuglycoside II are estimated at approximately 2.6% and 4.6%, respectively.

## 4. Conclusion

In this study, a liquid-liquid extraction method was employed to process rat plasma samples and ginsenoside Rg1 served as an internal standard for the establishment of a UPLC-MS/MS method to quantify ziyuglycoside I and ziyuglycoside II. The accuracy, precision, selectivity, and linearity of the method were validated. The developed UPLC-MS/MS method was applied to investigate the pharmacokinetics of ziyuglycoside I and ziyuglycoside II in rats following different administration routes (intragastric and intravenous), resulting in calculated bioavailabilities of 2.6% for ziyuglycoside I and 4.6% for ziyuglycoside II.

## Figures and Tables

**Table 1:** Accuracy, precision, matrix effect, and recovery of ziyuglycoside I and ziyuglycoside II in rat plasma.

Compound	Concentration (ng/mL)	Accuracy (%)		Precision (RSD%)		Matrix effect (%)	Recovery (%)
		Intra-day	Inter-day	Intra-day	Inter-day		
Ziyuglycoside I	2	89.7	108.6	10.2	7.7	109.6	84.6
	8	101.9	99.8	8.9	10.9	88.4	89.0
	150	96.0	104.6	9.2	4.8	112.5	88.1
	1500	98.0	97.0	4.2	4.8	109.7	94.4

Ziyuglycoside II	2	87.9	106.8	14.1	13.6	95.5	92.0
	8	100.9	98.4	5.9	9.0	98.2	89.0
	150	109.9	106.2	8.3	10.5	88.4	90.1
	1500	106.7	97.7	9.7	1.4	96.8	88.9

**Table 2:** Main pharmacokinetic parameters after intragastric **(**5 mg/kg) and intravenous (1 mg/kg) administration of ziyuglycoside I and ziyuglycoside II in rats.

Compound	Group	AUC_(0-*t*)_	AUC_(0-∞)_	*t*_1/2z_	CL_z/F_	*V*_z/F_	*C*_max_
		ng/mL *∗* h	ng/mL *∗* h	h	L/h/kg	L/kg	ng/mL
Ziyuglycoside I	ig	95.4 ± 7.2	109.0 ± 11.8	5.1 ± 2.5	46.3 ± 5.2	329.2 ± 143.6	36.6 ± 5.8
	iv	834.7 ± 249.2	838.3 ± 250.3	1.8 ± 0.7	1.3 ± 0.3	3.5 ± 2.0	1268.0 ± 344.8

Ziyuglycoside II	ig	438.9 ± 42.4	458.3 ± 46.3	4.9 ± 1.5	11.0 ± 1.0	77.3 ± 22.5	102.2 ± 14.5
	iv	1933.2 ± 209.1	1979.2 ± 185.7	6.2 ± 3.1	0.5	4.6 ± 2.4	1374.3 ± 224.5

## Data Availability

The data used to support the findings of this study are included within the article.
